# Beyond Hunger: Uncovering the Link between Food Insecurity and Depression, Anxiety, and Stress in Adolescents

**DOI:** 10.1016/j.cdnut.2025.107453

**Published:** 2025-04-28

**Authors:** Emily Cisneros-Vásquez, Lee Smith, Rodrigo Yañéz-Sepúlveda, Jorge Olivares-Arancibia, Héctor Gutiérrez-Espinoza, Dong Keon Yon, Jae Il Shin, José Francisco López-Gil

**Affiliations:** 1 School of Medicine, Universidad Espíritu Santo, Samborondón, Ecuador; 2 Centre for Health Performance and Wellbeing, Anglia Ruskin University, Cambridge, United Kingdom; 3 Biruni University, Faculty of Medicine, Department of Public Health, Instanbul, Turkey; 4 Faculty Education and Social Sciences, Universidad Andres Bello, Viña del Mar, Chile; 5 AFySE Group, Research in Physical Activity and School Health, School of Physical Education, Faculty of Education, Universidad de Las Américas, Santiago, Chile; 6 Faculty of Education, Universidad Autónoma de Chile, Santiago, Chile; 7 Center for Digital Health, Medical Science Research Institute, Kyung Hee University Medical Center, Kyung Hee University College of Medicine, Seoul, Republic of Korea; 8 Department of Pediatrics, Yonsei University College of Medicine, Seoul, Republic of Korea; 9 Vicerrectoría de Investigación y Postgrado, Universidad de Los Lagos, Osorno, Chile

**Keywords:** adolescents, anxiety, depression, food insecurity, mental health, stress

## Abstract

**Background:**

Food insecurity (FI) represents a critical public health concern, particularly for adolescents, as it compromises nutritional intake and mental health during crucial developmental stages.

**Objectives:**

This study examines the associations between FI and symptoms of depression, anxiety, and stress in a sample of 712 adolescents aged 12–17 y from *Valle de Ricote*, Region of Murcia, Spain.

**Methods:**

Data were sourced from the cross-sectional “Eating Healthy and Daily Life Activities” study. FI was assessed via the Child Food Security Survey Module, whereas mental health symptoms were evaluated via the Depression, Anxiety, and Stress Scale. Generalized linear models adjusted for socioeconomic status, lifestyle factors, and anthropometric variables were employed to estimate the relationships between FI and psychological outcomes.

**Results:**

Of the 712 adolescents (median age 14 y; 56% girls), 16.2% experienced FI. These adolescents had significantly greater risks of mental health symptoms: the likelihood of experiencing depression, anxiety, and stress was 2–3 times greater than that of their food-secure peers (odds ratios ranging from 2.45 to 3.35). Notably, the predicted probabilities of experiencing symptoms of anxiety and stress among food-insecure adolescents were 39.2% and 43.5%, respectively, whereas they were 16.1% and 19.8%, respectively, among their food-secure peers (*P* < 0.001 for both comparisons).

**Conclusions:**

These results underscore the profound psychological toll of FI and highlight the necessity of targeted interventions to address this issue. Addressing FI through public health policies and psychosocial programs is essential for mitigating its detrimental impact on adolescent mental health.

## Introduction

Mental health complications, including depression, anxiety, and stress, pose a significant challenge to adolescent well-being, impacting both physical and psychological health [1,2]. The WHO highlights that depression is one of the leading causes of morbidity and disability in this population, with a global prevalence of 14% [1]. Likewise, anxiety affects between 5% and 10% of adolescents worldwide [3]. Within the European Union, it is estimated that 10%–15% of young people experience mental health complications, with depression and anxiety being the most common [2]. In Spain, ∼5% of adolescents exhibit symptoms of depression and anxiety, whereas 62% report experiencing some related symptoms [4,5]. Psychological stress, although not always classified as a clinical disorder, is highly prevalent during this developmental stage and is often linked to psychosocial stressors such as academic pressure, social relationships, and family dynamics [6].

Food insecurity (FI) is a global public health issue with profound implications for adolescent health, particularly during critical growth and developmental stages [7]. FI refers to the lack of consistent access to sufficient, safe, and nutritious food, which negatively impacts dietary intake and overall well-being [8]. Adolescents are particularly vulnerable, as inadequate nutrition during this period can lead to stunted growth, cognitive impairments, poor academic performance, and an increased risk of chronic diseases and mental health complications [9]. Recent data indicate that 51.1% of adolescents worldwide experience moderate-to-severe FI [10]. In Spain, FI affected 13.3% of the population between 2020 and 2021 [11], with Instituto Nacional de Estadística data indicating that 6.9% of children and adolescents suffer poor nutrition [12]. In Catalonia, the FI prevalence among adolescents reached 18.3% [13], with 40.7% of families with children reporting reduced food quality and quantity [14].

FI has emerged as a critical determinant of mental health and is closely associated with depression, anxiety, and stress [15]. The uncertainty surrounding access to adequate and nutritious food often triggers substantial psychological distress, which is further intensified by the social stigma linked to nontraditional or informal methods of food acquisition [16,17]. According to the theory of psychosocial stress, prolonged exposure to adverse social and material conditions, such as FI, activates biological stress responses that, over time, may compromise mental health [18]. Similarly, the theory of relative deprivation posits that individuals who perceive themselves as disadvantaged compared with others may experience negative emotions such as shame and resentment, which contribute to psychological distress and the development of internalizing disorders [19]. This distress frequently manifests as feelings of shame, helplessness, and social isolation, which are directly associated with depressive symptomatology [20].

Adolescents are particularly vulnerable to these effects because of their heightened sensitivity to material and psychosocial factors during key stages of development [21]. During adolescence, individuals undergo significant biological (e.g., hormonal changes), psychological (e.g., identity formation), and social (e.g., peer integration and autonomy seeking) transformations. These transitions can increase emotional reactivity and reduce coping capacity, thereby amplifying the psychological impact of adverse experiences [[22], [23], [24]]. The stress induced by food uncertainty is a significant driver of anxiety and depression, disproportionately affecting socioeconomically disadvantaged groups [17,25]. Studies highlight that FI encompasses not only material deprivation but also significant social dimensions, including stigma and diminished self-worth, which further exacerbate mental health disparities [20,26].

Empirical research underscores the global prevalence of FI’s impact on mental health. A comprehensive multicountry study involving 48,401 adolescents revealed that moderate FI was associated with a 36% increase in the likelihood of depressive symptoms, whereas severe FI increased this likelihood by a striking 81% [10]. In Catalunya, Spain, adolescents grappling with FI reported significantly elevated stress levels compared with their food-secure peers, underscoring the emotional toll of insufficient access to nutritious food [13]. Similar trends are evident in other countries, with Ghanaian adolescents from FI households exhibiting 3-fold higher odds of depression than their food-secure counterparts [27]. Furthermore, findings from the United States [15], Canada [28], Indonesia [29], and India [30] consistently demonstrate that FI is linked to heightened rates of anxiety, depression, and psychological distress among adolescents.

Despite this growing body of international evidence, there is a notable gap in the literature specific to Spain. Existing research is geographically concentrated—mostly in urban regions such as Catalunya—and, to date, no studies have examined the simultaneous associations between FI and multiple mental health outcomes, such as anxiety, depression, and stress, in adolescents. Moreover, population-based studies conducted in rural or semirural areas, where both FI and limited access to mental health resources may be more prevalent, are lacking. This underscores the need for regionally focused research that can provide a more comprehensive understanding of how FI affects adolescent mental health across diverse Spanish contexts.

FI is a growing phenomenon that impacts both physical and mental health, particularly among vulnerable populations such as adolescents. Adolescence is a critical developmental stage during which material deprivation can negatively influence psychological well-being and emotional development, potentially leading to long-term mental health consequences. International evidence has linked FI to the emergence of depressive, anxious, and stress-related symptoms in children and adolescents; however, research on this topic in Spain remains limited. Given the increasing prevalence of mental health issues and increasing rates of FI in Spain, addressing this problem is both urgent and necessary. Failure to intervene early may result in lasting psychological, educational, and socioeconomic consequences, affecting not only individual life trajectories but also broader societal well-being. Therefore, this study aims to explore the relationships between FI and symptoms of depression, anxiety, and stress in Spanish adolescents aged 12–17 y. Understanding these associations is essential for developing targeted, effective psychosocial interventions that promote adolescent mental health and reduce health inequities in Spain.

## Methods

### Participants and study design

This study conducts a secondary analysis using data obtained from the cross-sectional study “Eating Healthy and Daily Life Activities (EHDLA),” which includes a representative sample of adolescents aged 12–17 y from the *Valle de Ricote* (Region of Murcia, Spain). A simple random sampling technique was employed to select participants for the EHDLA study. Data collection was carried out in 3 secondary education schools in the region from 2021 to 2022. The detailed methodological approach employed in the EHDLA study has been described in previous publications [31]. Participants were eligible for inclusion if they were aged 12–17 y and either registered or resided in Valle de Ricote. The exclusion criteria were as follows: *1*) exemption from physical education classes, as assessments and questionnaires were administered during these sessions; *2*) medical conditions contraindicating physical activity or requiring special care; *3*) ongoing pharmacological treatment; *4*) absence of parental or legal guardian consent; and *5*) the participant’s voluntary refusal to engage in the study.

Among the initial 1378 adolescents included in the EHDLA study, participants were excluded stepwise due to missing data on key study variables and covariates. First, 523 participants (38.0%) were excluded owing to the absence of Depression, Anxiety, and Stress Scale (DASS-21) scores, reducing the sample to 855. Next, 93 participants (6.7%) were removed due to incomplete Child Food Security Survey Module (CFSSM-S) data, resulting in 762 adolescents. A further 38 participants (2.8%) were excluded because BMI (in kg/m^2^) data were unavailable, reducing the sample to 724. Lastly, 12 participants (0.9%) were excluded due to missing Youth Activity Profile Physical (YAP-S) scores. The final analytic sample therefore consisted of 712 adolescents (Supplemental Figure 1).

### Study variables

#### Depression, anxiety, and stress (dependent variables)

The participants' symptoms of depression, anxiety, and stress were assessed via the validated Spanish version of the DASS-21 [32,33]. The DASS-21 consists of 21 items rated on a 3-point Likert scale ranging from 0 (did not apply to me at all) to 3 (applied to me very much or most of the time). The items are divided into 3 subscales, depression, anxiety, and stress, with the following cutoff points established: 6 points, 5 points, and 6 points, respectively [34].

#### FI (independent variable)

The assessment of FI was conducted via the CFSSM-S, which has been validated in Spanish [35]. This instrument evaluates participants' perceptions of FI within their households, addressing concerns such as risk of running out of food, consuming inexpensive food, difficulties in maintaining a balanced diet, the need to reduce portion sizes, skipping meals, experiencing hunger, or, in extreme cases, going an entire day without eating. The CFSSM-S consists of 9 items evaluated on a 3-point Likert scale, where affirmative responses (“often” and “sometimes”) are scored as 1 point and negative responses (“never”) are scored as 0 points. Food security (FS) classification was based on the criteria established by Connell et al. [36] and the USDA [37], categorizing households into the following groups: FS (0–1 points), low FS (2–5 points) and very low FS (6–9 points). However, owing to the low number of households in the “very low FS” category, the categories were recoded into 2 groups: “FS” and “FI.”

#### Covariates

The covariates were selected on the basis of their potential to influence the relationship between FI and mental health outcomes, specifically depression, anxiety, and stress. Age and sex were included because of their roles in psychological development and biological differences [16,21]. Socioeconomic status (SES) is considered because of its well-established association with both food FI and mental health [38]. Physical activity and sedentary behavior are lifestyle factors that can affect mental health [7]. Total sleep duration was considered due to its known impact on depression and anxiety [39], whereas total caloric intake was included, as it reflects dietary patterns that may interact with FI [40,41].

##### Anthropometric factors

Adolescents' body weight was measured via an electronic scale (Tanita BC-545), and height was measured via a portable stadiometer (Leicester Tanita HR 001). BMI was calculated by dividing weight (in kg) by height (in square meters) [31].

##### Sociodemographic factors

Data on sex and date of birth were self-reported by the participants. Age was calculated on the basis of birth date. SES was assessed via the Family Affluence Scale (FAS-III) [42]. The FAS-III score was derived by summing the responses to 6 questions related to car ownership, having room of one’s own, the number of computers in the household, the number of bathrooms (with a shower and/or bathtub), the availability of a dishwasher, and the frequency of travel outside Spain in the past year. The final score ranges from 0 to 13 points and is divided into 3 socioeconomic levels: low (0–2 points), medium (3–5 points), and high (≥6 points).

##### Lifestyle factors

Information regarding physical activity and sedentary behavior among adolescents was collected via the YAP-S tool, which has been validated for use in Spanish populations [43]. The YAP score is based on a self-administered 7-d questionnaire comprising 15 items rated on a 5-point Likert scale. The items are categorized into 3 domains: school activity, out-of-school activity, and sedentary behavior [44]. Scores for physical activity (in school and out of school) and sedentary behavior were calculated by summing the items within each domain.

Sleep duration (in minutes) was assessed by individually asking participants about their usual bedtime and wake-up times on weekdays and weekends. The average daily sleep duration for each participant was calculated via the following formula: [(average nocturnal sleep duration on weekends × 2) + (average nocturnal sleep duration on weekdays × 5)]/7.

Energy intake was estimated via a self-administered food frequency questionnaire validated for the Spanish population [45]. This instrument includes 45 items distributed across 12 food groups: red and processed meats; poultry, fish, and eggs; fruits; vegetables; dairy products; savory cereals; sweet cereals; legumes; nuts; sweets; sugar-sweetened beverages; and alcoholic beverages. To estimate total energy intake, the average weekly portion size for each food group was calculated by asking participants about their weekly and monthly consumption in each category.

### Statistical analysis

To assess the normality of the variables, visual techniques such as density plots and quantile‒quantile (Q‒Q) plots were employed, complemented by the Shapiro‒Wilk test. Quantitative variables were summarized using the median and IQR, whereas qualitative variables were described in terms of frequencies (*n*) and percentages (%). To examine the associations between FI and symptoms of depression, anxiety, and stress in adolescents, a generalized linear model (GLM) was used. These models apply robust methods to address heteroscedasticity and outliers. An a priori power analysis was performed to estimate the minimum sample size needed to detect a moderate effect size (*R*^2^ = 0.13) in a GLM with 9 predictors, using a conventional alpha level (*α* = 0.05) and a statistical power of 80% (1−*β* = 0.80). The analysis indicated that a minimum of 114 participants would be necessary to achieve adequate power. Because the current study included a sample of 712 adolescents, the statistical power exceeded the required threshold, ensuring sufficient sensitivity to detect meaningful associations. For continuous outcomes, a GLM with a Gaussian distribution was implemented via the “*SMDM*” method (involving an S estimation followed by an M estimation, an adaptive scale design estimation, and another M step). For dichotomous outcomes, GLMs with a binomial distribution and the “*Mqle*” method were employed. Additionally, the estimated marginal means (M) of the DASS-21 score (which reflects symptoms of depression, anxiety, or stress) or the predictive probabilities (%) for each symptom, along with their 95% confidence intervals (CIs), were calculated on the basis of FI. All models were adjusted for various covariates, such as sex, age, socioeconomic level, total sleep duration, physical activity, sedentary behavior, and energy intake. Statistical analyses were performed via R software (version 4.3.2; R Core Team) with RStudio (version 2023.12.1 + 402; Posit). A 2-sided *P* value < 0.05 was considered significant for all the statistical analyses.

## Results

Table 1 presents the descriptive data of the participants (*n =* 712), categorized according to their FI status. Among the total sample, 16.2% (*n =* 115) experienced FI. An analysis of the demographic variables revealed a similar sex distribution across both groups, with a slight predominance of females in each group (*P <* 0.001). The median age was consistent between the groups, at 14 y (IQR = 2.0). However, a minor difference was noted in the scores of the FAS-III, with slightly lower values in the FI group (median = 7.0, IQR = 3.5) than in the FS group (median = 8.0, IQR = 3.0). The mean BMI in the FI group was slightly greater than that in the FS group (22.1 compared with 21.6). The additional analysis confirmed that BMI was not significantly associated with symptoms of depression, anxiety, or stress in adolescents (*P =* 0.579, *P =* 0.365, and *P =* 0.411, respectively; see Supplemental Table 1). Furthermore, the FI group reported greater average energy intake (3142.4 kcal compared with 2498.9 kcal in the FS group). Other parameters, such as total sleep duration, physical activity level, and sedentary behavior, showed minimal differences between the groups.

**TABLE 1:** Descriptive data of the study participants according to food insecurity status (*n =* 712)

Variable		Food insecurity status	
		Food security	Food insecurity
Participants	*n* (%)	597 (83.8)	115 (16.2)
Sex	Boys	262 (43.9)	51 (44.3)
	Girls	335 (56.1)	64 (55.7)
Age (y)	Median (IQR)	14.0 (2.0)	14.0 (2.0)
FAS-III (score)	Median (IQR)	8.0 (3.0)	7.0 (3.5)
BMI (kg/m^2^)	Median (IQR)	21.6 (5.9)	22.1 (6.7)
Overall sleep duration (min)	Median (IQR)	501.4 (68.6)	501.4 (77.1)
YAP-S physical activity (score)	Median (IQR)	2.6 (0.8)	2.7 (0.9)
YAP-S sedentary behaviors (score)	Median (IQR)	2.4 (0.8)	2.6 (0.9)
Energy intake (kcal)	Median (IQR)	2498.9 (1364.8)	3142.4 (2326.7)
DASS-21 depression (score)	Median (IQR)	2.0 (6.0)	5.0 (9.0)
Depression (%)	No (%)	437 (73.2)	60 (52.2)
	Yes (%)	160 (26.8)	55 (47.8)
DASS-21 anxiety (score)	Median (IQR)	2.0 (6.0)	6.0 (9.0)
Anxiety (%)	No (%)	422 (70.7)	50 (43.5)
	Yes (%)	175 (29.3)	65 (56.5)
DASS-21 stress (score)	Median (IQR)	4.0 (7.0)	6.0 (8.0)
Stress (%)	No (%)	387 (64.8)	47 (40.9)
	Yes (%)	210 (35.2)	68 (59.1)

Abbreviations: DASS-21, Depression, Anxiety and Stress Scale—21 Items; FAS-III, Family Affluence Scale-III; YAP-S, Spanish Youth Activity Profile.

The analyses revealed significant differences between adolescents experiencing FI and those experiencing FS conditions concerning the 3 dimensions assessed by the DASS-21. Specifically, compared with their FS counterparts, adolescents with FI presented higher median scores on the depression (5.0 compared with 2.0), anxiety (6.0 compared with 2.0), and stress (6.0 compared with 4.0) subscales. Additionally, when classified by the level of each condition, the percentages of adolescents with symptoms of depression (47.8%), anxiety (56.5%), and stress (59.1%) were considerably greater in the FI group than in the FS group (26.8%, 29.3%, and 35.2%, respectively).

**FIGURE 1:**
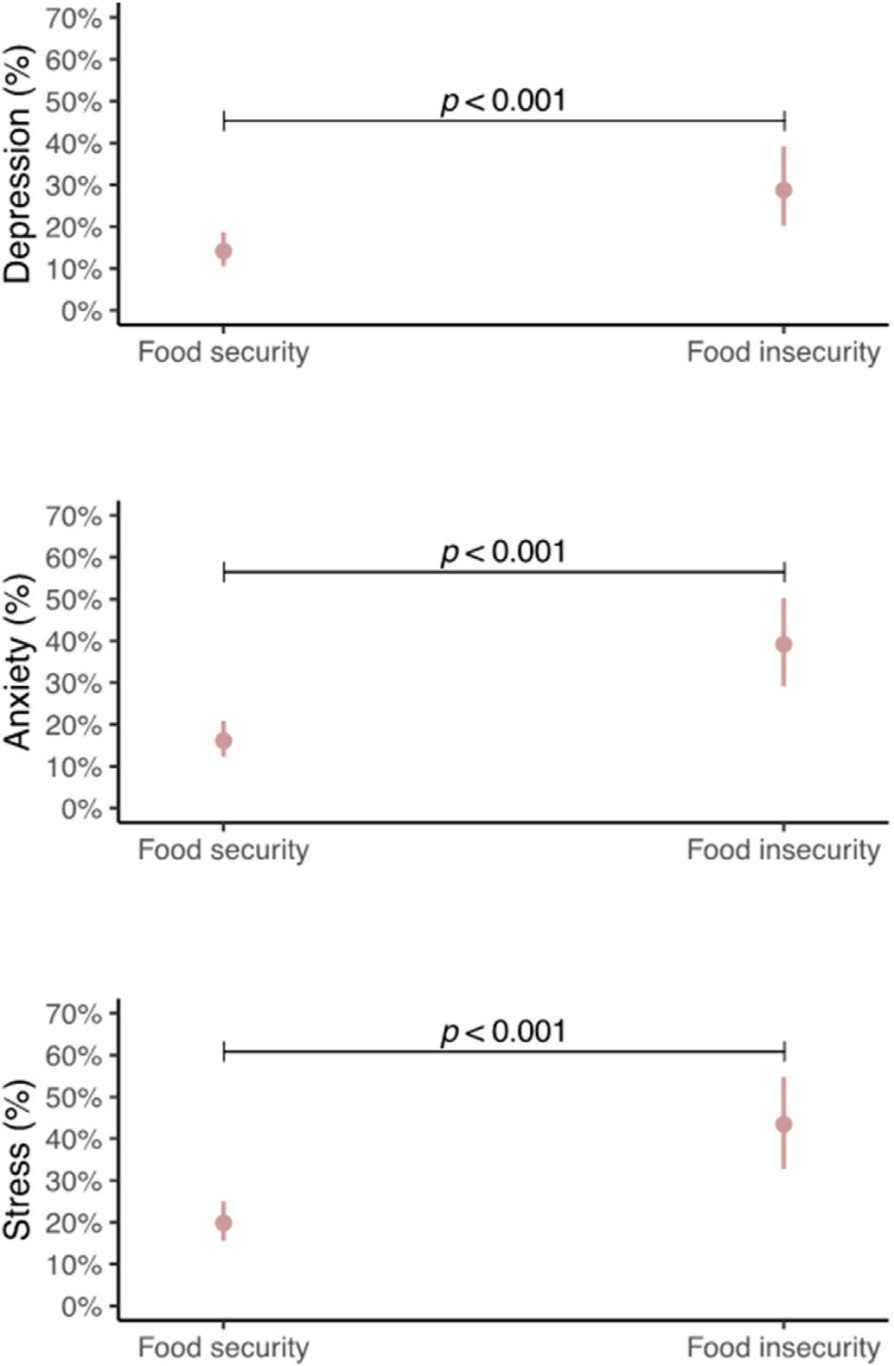
Predictive probabilities of having depression, anxiety, and stress on the basis of food insecurity status among adolescents. Adjusted for age, sex, socioeconomic status, physical activity, sedentary behavior, sleep duration, BMI, and energy intake.

The predictive probabilities of experiencing symptoms of depression, anxiety, and stress are markedly greater among adolescents facing FI, as shown in Table 2. Specifically, these adolescents exhibited a significantly greater likelihood of presenting symptoms of depression (28.8% compared with 14.2%), anxiety (39.2% compared with 16.1%), and stress (43.5% compared with 19.8%), with statistically significant differences (*P <* 0.001), as illustrated in Figure 1. The odds ratios (ORs) indicate that FI substantially increases risk of suffering from depression (OR = 2.45, *P <* 0.001, 95% CI: 1.55, 3.85), anxiety (OR = 3.35, *P <* 0.001, 95% CI: 2.14, 5.26), and stress (OR = 3.10, *P <* 0.001, 95% CI: 1.97, 4.91) (see Supplemental Table 2). The full results of the GLMs are detailed in Supplemental Table 1, Supplemental Table 2, Supplemental Table 3, and Supplemental Figure 2.

**TABLE 2:** Predictive probabilities of having depression, anxiety, and stress among adolescents

Outcome	Food security	Food insecurity
	% (95% CI)	% (95% CI)
Depression^1^	14.2 (10.5, 18.8)	28.8 (20.2, 39.2)^2^
Anxiety^1^	16.1 (12.3, 20.9)	39.2 (29.1, 50.3)^2^
Stress^1^	19.8 (15.6, 25.0)	43.5 (32.8, 54.7)^2^

Abbreviations: %, predictive probabilities; CI, confidence interval.

1 According to the DASS-21, Depression, Anxiety and Stress Scale—21 items.

2 Significant difference from food security status (*P <* 0.001).

## Discussion

### Main findings

The present analysis uses data from the EHDLA study and explores the associations between FI and mental health complications in adolescents aged 12–17 y from the Valle de Ricote, Region of Murcia, Spain. These findings indicate that, compared with their counterparts with FS, adolescents experiencing FI are at a considerably greater risk of exhibiting symptoms of depression, anxiety, and stress. Using the DASS-21 scale, the study revealed significantly elevated scores across all domains among participants with FI. Furthermore, the ORs demonstrated that FI increased the likelihood of depression, anxiety, and stress-related symptoms by ∼2 to 3 times. These results underscore the detrimental psychological impact of FI during adolescence, a critical period of emotional and cognitive development. The implications of these findings are essential for informing public health policies and targeted interventions aimed at reducing the mental health burden associated with socioeconomic vulnerability in young populations.

### Comparison with previous studies

Our findings align significantly with studies conducted in other international contexts, highlighting the relationship between FI and increased symptoms of depression, anxiety, and stress among adolescents. In Catalunya, Spain, a study using a similar method to measure FI (CFSSM-S) also revealed a higher prevalence of stress among adolescents experiencing FI, although the results focused on stress and did not specifically assess anxiety and depression [13]. In Ghana [27] and India [30], these associations are particularly pronounced, with adolescents from FI households being ≤3 times more likely to experience depression or high levels of psychological distress. These findings are consistent with our findings but reveal a higher prevalence of FI, which may reflect significant socioeconomic differences. In Canada [28] and Indonesia [29], associations between FI and symptoms of depression and anxiety were also consistent; however, the tools employed (CBCL, K10) and the inclusion of broader age ranges may have influenced the variability of the results. Differences in evaluation methods and geographic contexts underscore the need for contextualized approaches to address the relationship between FI and adolescent mental health.

### Nutritional deficiencies

Nutritional deficiencies are a significant mechanism through which FI may contribute to the development of mental health disorders. Insufficient intake of essential nutrients, such as polyunsaturated fatty acids, proteins, and key micronutrients (e.g., zinc, copper, and magnesium), has been associated with increased vulnerability to psychological distress [46]. Low levels of omega-3 fatty acids, for instance, are linked to increased anxiety, whereas deficiencies in vitamins from the B-complex disrupt neurotransmitter synthesis, impairing serotonin, dopamine, and norepinephrine production, which are critical for mood regulation [40]. Furthermore, diets lacking selenium, calcium, and vitamin C have been correlated with poor sleep quality, a known contributor to mental health issues [41].

Although adolescents experiencing FI reported a higher average energy intake than their FS peers did (3142.4 kcal compared with 2498.9 kcal), this does not imply better dietary quality. This elevated caloric intake may be driven by the consumption of inexpensive, energy-dense, nutrient-poor foods, such as ultraprocessed products rich in saturated fats and added sugars. Although this study did not include a detailed assessment of nutrient intake, prior research has indicated that deficiencies in key nutrients, such as omega-3 fatty acids, B vitamins, and minerals such as zinc and magnesium, may heighten risk of mental health disorders [46]. The lack of specific dietary data limits definitive conclusions; however, these findings underscore the importance of future studies incorporating comprehensive nutritional assessments to clarify the role of dietary inadequacies in the association between FI and mental health among adolescents. Furthermore, chronic FI has been shown to elevate cortisol levels and stress reactivity, compounding psychological distress [47]. Thus, addressing nutritional deficits in food-insecure populations may be a critical component of mental health prevention and intervention strategies.

### Social stigmatization

Social stigma plays a pivotal role in linking FI to the development of psychological issues in adolescents. Those experiencing FI often face societal judgment and discrimination, stemming from stereotypes that are associated with their struggles with personal failure or parental inadequacy [48]. These stigmatizing perceptions foster shame, embarrassment, and low self-worth, especially when socioeconomic challenges are visible to others [49]. Consequently, adolescents may withdraw from social interactions to avoid judgment, resulting in isolation and heightened emotional distress [17]. Although our study did not directly assess adolescents’ experiences of stigma, the observed higher prevalence of depression, anxiety, and stress among adolescents with FI suggests the potential contribution of psychosocial mechanisms, including stigmatization. Chronic exposure to stigma exacerbates stress responses and fosters a sense of helplessness and hopelessness, which are key features of depression [6]. Moreover, the fear of being labeled or excluded intensifies anxiety and undermines the willingness to seek support, perpetuating poor mental health outcomes [48]. Mitigating these impacts requires addressing FI beyond nutrition access by reducing societal stigma through education and fostering inclusive, empathetic environments.

### Sleep disturbances

FI has been identified as a potential risk factor for the emergence of depression, stress, and anxiety among adolescents, with sleep disturbances serving as one of the mechanisms through which this relationship operates [50]. Insufficient or poor-quality sleep is commonly observed among adolescents experiencing FI, as they may face physiological stressors related to inadequate nutrition or psychological stress stemming from the uncertainty surrounding access to food [51]. Studies suggest that sleep deprivation and irregular sleep patterns can disrupt the regulation of mood and stress responses, exacerbating symptoms of anxiety and depression [39]. Moreover, the link between FI and disrupted sleep may further amplify the emotional and cognitive challenges faced by adolescents, creating a vicious cycle [46]. Adolescents with irregular sleep are more likely to experience irritability, fatigue, and difficulty concentrating, all of which contribute to risk of developing mental health issues [39].

The present study revealed no significant differences in total sleep duration between adolescents with and without FI, with both groups reporting an identical average of 501.4 min per night. However, sleep duration alone does not capture key aspects such as sleep quality, continuity, or subjective satisfaction. Research indicates that even if sleep duration is adequate, poor sleep quality can still lead to negative health outcomes, including impaired cognitive function and emotional instability [[52], [53], [54]]. Thus, although no differences in sleep quantity were observed, future investigations should incorporate validated tools to assess sleep quality and disturbances to further explore this potential mediating pathway.

### Strengths and limitations

This study has limitations that must be acknowledged. The cross-sectional design limits the ability to infer causality between FI and the psychological outcomes of depression, anxiety, and stress, as it does not establish whether FI contributes to poor mental health or whether pre-existing mental health conditions increase risk of FI. Mental health complications, such as impaired cognitive function, reduced motivation, and difficulty maintaining employment, may contribute to FI by limiting an individual's capacity to secure a stable income and access nutritious food [55]. The reliance on self-reported measures, including the DASS-21 and CFSSM-S, may introduce reporting bias due to memory errors or social desirability. Furthermore, the exclusion of 48.3% of the initial sample due to missing data may introduce selection bias, particularly if the missing data are not random. This bias could, in turn, limit the generalizability of the findings to the target population. Nevertheless, the study has notable strengths, such as the use of validated tools and comprehensive adjustment for covariates, including SES, lifestyle behaviors, and anthropometric factors. These findings underscore the critical public health implications of addressing FI as a modifiable determinant of mental health. Policies aimed at providing economic support and ensuring access to nutritious food for food-insecure adolescents are essential. Future longitudinal research is warranted to elucidate the causal pathways and long-term mental health consequences of FI in this vulnerable population.

In conclusion, this study highlights the significant associations between FI and increased symptoms of depression, anxiety, and stress among adolescents in Valle de Ricote, Murcia, Spain. Adolescents facing FI were 2–3 times more likely to report these symptoms, emphasizing the psychological burden of limited access to nutritious food during a critical stage of emotional and cognitive development. These findings are consistent with international research, underscoring FI as a modifiable risk factor with broader implications for adolescent mental health. The likely mechanisms underlying this association include nutritional deficiencies, sleep disturbances, and social stigma, which together perpetuate a cycle of vulnerability.

In addition to its theoretical contributions, this study offers actionable insights for clinical, educational, and social practice. In clinical settings, integrating routine nutritional assessments into adolescent mental health evaluations may facilitate early identification of those at risk and promote timely psychological support. In schools, implementing nutritional support programs (e.g., free meals) alongside workshops on emotional regulation could enhance both physical and mental well-being. From a public health perspective, these results reinforce the need for policies that guarantee equitable access to nutritious food and promote awareness of the nutritional–mental health link. Concrete strategies, such as school-based screenings with tools such as the DASS-21, group cognitive‒behavioral interventions in health centers, and municipal food aid initiatives, could be coordinated across sectors to reduce the adverse effects of FI. Ultimately, addressing FI through multisectoral interventions may help safeguard adolescent mental health, fostering resilience and long-term well-being.

## Author contributions

The authors’ responsibilities were as follows – JFL-G: designed the research, conducted the research, resources, analyzed the data, data curation, visualization, supervision, and project administration; EC-V: writing—original draft preparation; and all authors: writing—review and editing, read and approved the final manuscript.

## Institutional review board statement

This study obtained ethical approval from the Bioethics Committee of the University of Murcia (ID 2218/2018; approval date: 18 February, 2019), as well as the Ethics Committee of the *Complejo Hospitalario Universitario de Albacete* and the *Gerencia de Atención Integrada de Albacete* (ID 2021-85; approval date: 23 November, 2021). The research adhered to the principles of the Declaration of Helsinki, ensuring the protection of human rights for all participants.

## Informed consent statement

Informed consent was obtained from all the subjects involved in the study.

## Data availability statement

Data described in the manuscript, code book, and analytic code will be made available on request pending.

## Funding

This research received no external funding.

## Conflict of interest

The authors declare that they have no conflicts of interest.
