# Corrigendum to “Beyond Hunger: Uncovering the Link between Food Insecurity and Depression, Anxiety, and Stress in Adolescents [Current Developments in Nutrition 9 (2025) 107453]

**DOI:** 10.1016/j.cdnut.2025.107570

**Published:** 2025-10-10

**Authors:** Emily Cisneros-Vásquez, Lee Smith, Rodrigo Yañéz-Sepúlveda, Jorge Olivares-Arancibia, Héctor Gutiérrez-Espinoza, Dong Keon Yon, Jae Il Shin, José Francisco López-Gil

**Affiliations:** 1 One Health Research Group, Universidad de Las Américas, Quito, Ecuador; 2 School of Medicine, Universidad Espíritu Santo, Samborondón, Ecuador; 3 Centre for Health Performance and Wellbeing, Anglia Ruskin University, Cambridge, United Kingdom; 4 Biruni University, Faculty of Medicine, Department of Public Health, Istanbul, Turkey; 5 Faculty Education and Social Sciences, Universidad Andres Bello, Vina del Mar, Chile; 6 AFySE Group, Research in Physical Activity and School Health, School of Physical Education, Faculty of Education, Universidad de Las Américas, Santiago, Chile; 7 Faculty of Education, Universidad Autónoma de Chile, Santiago, Chile; 8 Center for Digital Health, Medical Science Research Institute, Kyung Hee University Medical Center, Kyung Hee University College of Medicine, Seoul, Republic of Korea; 9 Department of Pediatrics, Yonsei University College of Medicine, Seoul, Republic of Korea; 10 Vicerrectoría de Investigación y Postgrado, Universidad de Los Lagos, Osorno, Chile

Emily Cisneros-Vásquez and José Francisco López-Gil regret that their Universidad de Las Américas affiliation was inadvertently omitted in the original publication of the article. This corrigendum updates the article to reflect correct affiliations. The authors would like to apologize for any inconvenience caused.

