# Supplementary material for: Beyond Hunger: Uncovering the Link between Food Insecurity and Depression, Anxiety, and Stress in Adolescents
Source: Curr Dev Nutr. 2025 Apr 28;9(6):107453. doi: 10.1016/j.cdnut.2025.107453 (PMC12148393; doi:10.1016/j.cdnut.2025.107453)
Supplement: multimedia component 1 [file mmc1.docx]

## Supplementary material

# Supplementary Table 1. Association between food insecurity status and symptoms of depression, anxiety, and stress among adolescents.

|  | **Outcome: DASS-21 Depression (score)** | | | | |
| --- | --- | --- | --- | --- | --- |
| **Predictor** | ***B*** | **SE** | **95% LLCI** | **95% ULCI** | ***p*-value** |
| Food security | Reference |  |  |  |  |
| Food insecurity | 2.43 | 0.45 | 1.56 | 3.30 | <0.001 |
| Age | -0.03 | 0.11 | -0.25 | 0.18 | 0.768 |
| Boys | Reference |  |  |  |  |
| Girls | 2.43 | 0.33 | 1.78 | 3.07 | <0.001 |
| FAS-III score (per one point) | -0.17 | 0.08 | -0.32 | -0.02 | 0.026 |
| BMI (per one kg/m^2^) | 0.02 | 0.03 | -0.05 | 0.08 | 0.579 |
| Overall sleep duration (per one hour) | -0.60 | 0.19 | -0.97 | -0.22 | 0.002 |
| YAP-S physical activity (per one point) | 0.05 | 0.25 | -0.43 | 0.53 | 0.841 |
| YAP-S sedentary behaviors (per one point) | 0.60 | 0.28 | 0.05 | 1.16 | 0.034 |
| Energy intake (per 1000 kcal) | -0.07 | 0.08 | -0.23 | 0.10 | 0.426 |
|  | **Outcome: DASS-21 Anxiety (score)** | | | | |
| **Predictor** | ***B*** | **SE** | **LLCI** | **ULCI** | ***p*-value** |
| Food security | Reference |  |  |  |  |
| Food insecurity | 2.51 | 0.42 | 1.68 | 3.33 | <0.001 |
| Age | -0.10 | 0.10 | -0.30 | 0.10 | 0.335 |
| Boys | Reference |  |  |  |  |
| Girls | 2.21 | 0.31 | 1.61 | 2.82 | <0.001 |
| FAS-III score (per one point) | -0.07 | 0.07 | -0.21 | 0.07 | 0.309 |
| BMI (per one kg/m^2^) | 0.03 | 0.03 | -0.03 | 0.09 | 0.365 |
| Overall sleep duration (per one hour) | -0.40 | 0.18 | -0.75 | -0.04 | 0.028 |
| YAP-S physical activity (per one point) | 0.03 | 0.23 | -0.42 | 0.49 | 0.888 |
| YAP-S sedentary behaviors (per one point) | 0.37 | 0.27 | -0.15 | 0.89 | 0.164 |
| Energy intake (per 1000 kcal) | 0.03 | 0.08 | -0.12 | 0.18 | 0.676 |
|  | **Outcome: DASS-21 Stress (score)** | | | | |
| **Predictor** | ***B*** | **SE** | **LLCI** | **ULCI** | ***p*-value** |
| Food security | Reference |  |  |  |  |
| Food insecurity | 2.21 | 0.47 | 1.29 | 3.13 | <0.001 |
| Age | 0.09 | 0.12 | -0.14 | 0.32 | 0.453 |
| Boys | Reference |  |  |  |  |
| Girls | 2.86 | 0.35 | 2.17 | 3.54 | <0.001 |
| FAS-III score (per one point) | -0.07 | 0.08 | -0.23 | 0.08 | 0.361 |
| BMI (per one kg/m^2^) | 0.03 | 0.04 | -0.04 | 0.10 | 0.411 |
| Overall sleep duration (per one hour) | -0.56 | 0.20 | -0.96 | -0.17 | 0.006 |
| YAP-S physical activity (per one point) | -0.12 | 0.26 | -0.64 | 0.40 | 0.647 |
| YAP-S sedentary behaviors (per one point) | 0.09 | 0.30 | -0.49 | 0.68 | 0.754 |
| Energy intake (per 1000 kcal) | 0.02 | 0.08 | -0.14 | 0.19 | 0.792 |

**Abbreviations**: *B*, unstandardized beta coefficient; BMI, body mass index; FAS-III, Family Affluence Scale-III; LLCI, lower limit confidence interval; SE, standard error; ULCI, upper limit confidence interval; YAP-S, Spanish Youth Activity Profile.

# Supplementary Table 2. Association between food insecurity status and depression, anxiety, and stress status among adolescents.

|  | **Outcome: DASS-21 Depression (status)** | | | | |
| --- | --- | --- | --- | --- | --- |
| **Predictor** | **OR** | **SE** | **95% LLCI** | **95% ULCI** | ***p*-value** |
| Food security | Reference |  |  |  |  |
| Food insecurity | **2.45** | **0.23** | **1.55** | **3.85** | **<0.001** |
| Age | 0.93 | 0.06 | 0.82 | 1.05 | 0.230 |
| Boys | Reference |  |  |  |  |
| Girls | 3.52 | 0.20 | 2.38 | 5.21 | <0.001 |
| FAS-III score (per one point) | 0.95 | 0.04 | 0.87 | 1.03 | 0.196 |
| BMI (per one kg/m^2^) | 1.00 | 0.02 | 0.97 | 1.04 | 0.879 |
| Overall sleep duration (per one hour) | 0.78 | 0.11 | 0.63 | 0.95 | 0.016 |
| YAP-S physical activity (per one point) | 0.90 | 0.14 | 0.69 | 1.19 | 0.472 |
| YAP-S sedentary behaviors (per one point) | 1.27 | 0.16 | 0.93 | 1.73 | 0.133 |
| Energy intake (per 1000 kcal) | 0.98 | 0.04 | 0.90 | 1.06 | 0.576 |
|  | **Outcome: DASS-21 Anxiety (status)** | | | | |
| **Predictor** | **OR** | **SE** | **LLCI** | **ULCI** | ***p*-value** |
| Food security | Reference |  |  |  |  |
| Food insecurity | **3.35** | **0.23** | **2.14** | **5.26** | **<0.001** |
| Age | 0.97 | 0.06 | 0.86 | 1.09 | 0.596 |
| Boys | Reference |  |  |  |  |
| Girls | 3.45 | 0.19 | 2.38 | 5.02 | <0.001 |
| FAS-III score (per one point) | 0.99 | 0.04 | 0.91 | 1.07 | 0.828 |
| BMI (per one kg/m^2^) | 1.03 | 0.02 | 0.99 | 1.07 | 0.124 |
| Overall sleep duration (per one hour) | 0.91 | 0.10 | 0.74 | 1.11 | 0.349 |
| YAP-S physical activity (per one point) | 0.97 | 0.14 | 0.74 | 1.27 | 0.821 |
| YAP-S sedentary behaviors (per one point) | 1.03 | 0.15 | 0.76 | 1.39 | 0.850 |
| Energy intake (per 1000 kcal) | 1.00 | 0.04 | 0.92 | 1.09 | 0.940 |
|  | **Outcome: DASS-21 Stress (status)** | | | | |
| **Predictor** | **OR** | **SE** | **LLCI** | **ULCI** | ***p*-value** |
| Food security | Reference |  |  |  |  |
| Food insecurity | **3.10** | **0.23** | **1.97** | **4.91** | **<0.001** |
| Age | 0.96 | 0.06 | 0.86 | 1.08 | 0.484 |
| Boys | Reference |  |  |  |  |
| Girls | 3.46 | 0.18 | 2.43 | 4.94 | <0.001 |
| FAS-III score (per one point) | 1.02 | 0.04 | 0.94 | 1.11 | 0.609 |
| BMI (per one kg/m^2^) | 1.03 | 0.02 | 1.00 | 1.07 | 0.092 |
| Overall sleep duration (per one hour) | 0.80 | 0.10 | 0.66 | 0.98 | 0.031 |
| YAP-S physical activity (per one point) | 0.86 | 0.13 | 0.66 | 1.11 | 0.249 |
| YAP-S sedentary behaviors (per one point) | 0.83 | 0.15 | 0.62 | 1.12 | 0.220 |
| Energy intake (per 1000 kcal) | 0.99 | 0.04 | 0.91 | 1.08 | 0.851 |

**Abbreviations**: BMI, body mass index; FAS-III, Family Affluence Scale-III; LLCI, lower limit confidence interval; OR, odds ratio; SE, standard error; ULCI, upper limit confidence interval; YAP-S, Spanish Youth Activity Profile.

## Supplementary Table 3. Estimated marginal means of symptoms of depression, anxiety, and stress among adolescents.

| **Outcome** | **Food security** | **Food insecurity** |
| --- | --- | --- |
|  | **M (95% CI)** | **M (95% CI)** |
| Depression symptoms ^‡^ | 2.0 (1.5 to 2.4) | 4.4 (3.5 to 5.3)^†^ |
| Anxiety symptoms ^‡^ | 1.9 (1.5 to 2.4) | 4.4 (3.6 to 5.3)^†^ |
| Stress symptoms ^‡^ | 2.9 (2.3 to 3.4) | 5.1 (4.2 to 6.0)^†^ |

**Abbreviations**: CI, confidence interval; M, estimated marginal mean.

^†^ Significant differences from food security status (*p*<0.001).

^‡^ According to the DASS-21, Depression, Anxiety and Stress Scale - 21 Items.

**Supplementary Figure 1.** Flowchart of the selection process of the final study sample.

**
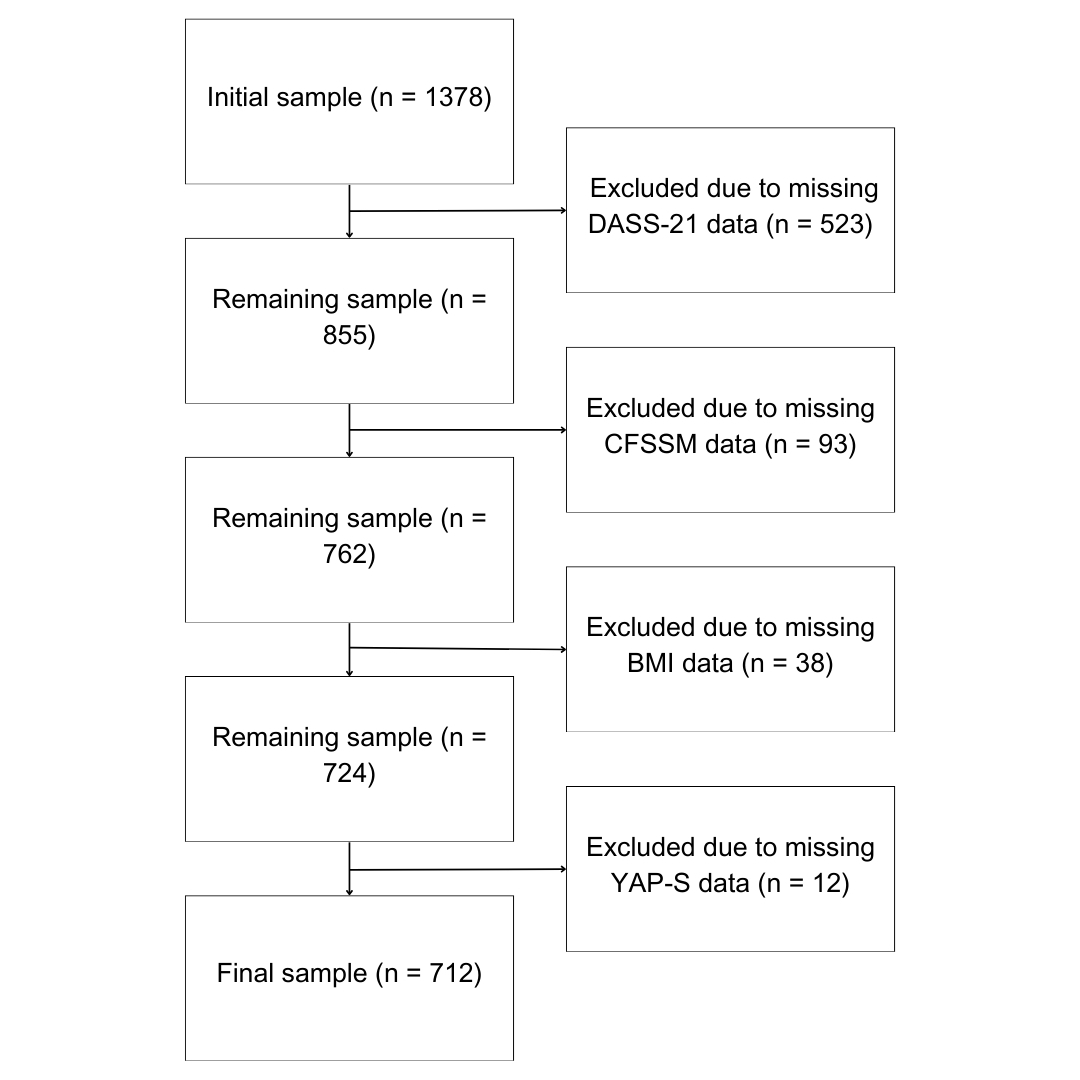
**

**Supplementary Figure 2.** Estimated marginal means of symptoms of depression, anxiety, and stress among adolescents. DASS-21, Depression, Anxiety and Stress Scale - 21 Items.

#
